# 2-Methyl­benzimidazolium nitrate

**DOI:** 10.1107/S1600536810008615

**Published:** 2010-03-13

**Authors:** Qingshuang Ma, Wenzeng Duan, Yudao Ma, Xiao Liu, Bo Qu

**Affiliations:** a School of Chemistry and Chemical Engineering, Shandong University, Jinan 250100, People’s Republic of China; b Department of Chemistry and Environmental Science, Taishan University, 271021 Taian, Shandong, People’s Republic of China

## Abstract

In the title compound, C_8_H_9_N_2_^+^·NO_3_^−^, inter­molecular N—H⋯O hydrogen bonds join the mol­ecules into a chain extending along the *b* axis.

## Related literature

For the applications of related benzimidazole compounds, see: Wright (1951 ▶); El-masry *et al.* (2000 ▶); Gümüş *et al.* (2003 ▶).
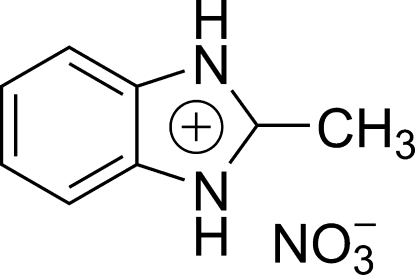


## Experimental

### 

#### Crystal data

C_8_H_9_N_2_^+^·NO_3_^−^
*M* *_r_* = 195.18
Monoclinic, 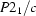

*a* = 7.711 (4) Å
*b* = 15.127 (7) Å
*c* = 8.270 (4) Å
β = 99.398 (7)°
*V* = 951.7 (8) Å^3^
*Z* = 4
Mo *K*α radiation
μ = 0.11 mm^−1^
*T* = 298 K
0.18 × 0.16 × 0.12 mm

#### Data collection

Bruker SMART APEX diffractometer
Absorption correction: multi-scan (*SADABS*; Bruker, 2005 ▶) *T*_min_ = 0.981, *T*_max_ = 0.987
4774 measured reflections
1685 independent reflections
1319 reflections with *I* > 2σ(*I*)
*R*_int_ = 0.027

#### Refinement

*R*[*F* ^2^ > 2σ(*F* ^2^)] = 0.045
*wR*(*F* ^2^) = 0.135
*S* = 1.07
1685 reflections
128 parameters
H-atom parameters constrained
Δρ_max_ = 0.22 e Å^−3^
Δρ_min_ = −0.21 e Å^−3^

### 

Data collection: *SMART* (Bruker, 2005 ▶); cell refinement: *SAINT* (Bruker, 2005 ▶); data reduction: *SAINT*; program(s) used to solve structure: *SHELXS97* (Sheldrick, 2008 ▶); program(s) used to refine structure: *SHELXL97* (Sheldrick, 2008 ▶); molecular graphics: *SHELXTL* (Sheldrick, 2008 ▶); software used to prepare material for publication: *SHELXL97*.

## Supplementary Material

Crystal structure: contains datablocks I, global. DOI: 10.1107/S1600536810008615/gk2255sup1.cif

Structure factors: contains datablocks I. DOI: 10.1107/S1600536810008615/gk2255Isup2.hkl

Additional supplementary materials:  crystallographic information; 3D view; checkCIF report

## Figures and Tables

**Table 1:** Hydrogen-bond geometry (Å, °)

*D*—H⋯*A*	*D*—H	H⋯*A*	*D*⋯*A*	*D*—H⋯*A*
N2—H2⋯O1^i^	0.86	2.03	2.855 (3)	162
N3—H3⋯O2^ii^	0.86	1.93	2.775 (2)	166

Symmetry codes: (i) 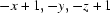; (ii) 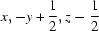.

**Fig. 1.**
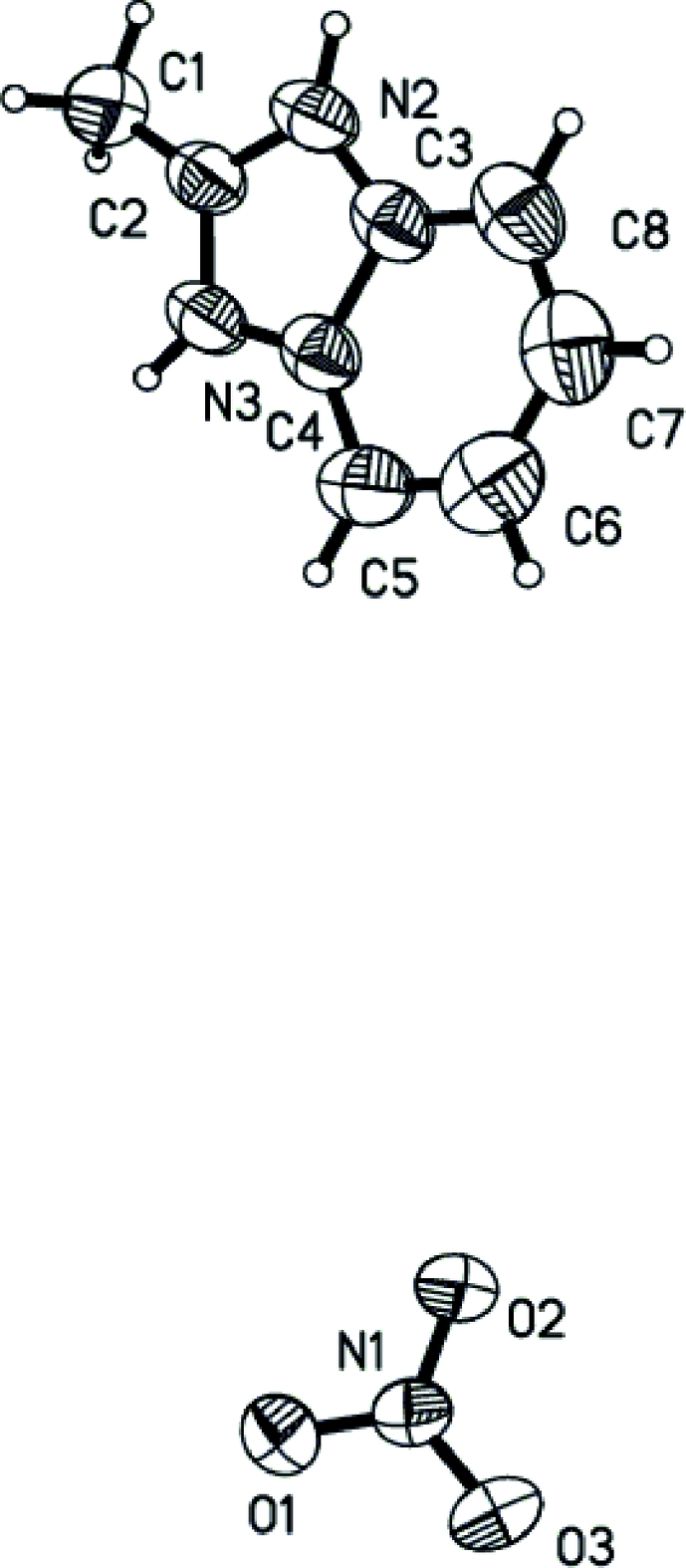
The structure of the title compound showing 50% probability displacement.

**Fig. 2.**
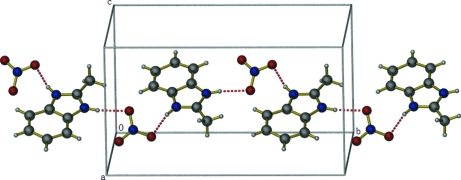
The supramolecular chain of the title compound formed via N—H···O hydrogen bonds.

## supplementary crystallographic information

### Comment

Benzimidazole and its derivatives have found practical applications in a number of fields (Wright, 1951). This ring system is present in numerous antiparasitic, antihelmintic and anti-inflammatory drugs (El-masry *et al.*, 2000). The complexes of transition metals with benzimidazole and related ligands have been extensively studied as models of some important biological molecules (Gümüş *et al.*, 2003). During our search to find new benzimidazole-metal complexes 2-methylbenzimidazole nitrate was unintentionally obtained.

Herein, we report the structure of the title compound, C_8_H_9_N_3_O_3 _(Fig 1). The crystal structure showed that intermolecular N—H···O hydrogen bonds link the molecules into a 1D polymeric structure (Fig. 2).

### Experimental

A mixture of *o*-phenylenediamine(1.08 g, 10 mmol) and anhydrous sodium acetate (2.46 g, 30 mmol) were dissolved in 100 mL 5% hydrochloric acid. After stirring for 2 h under reflux, the solution was cooled to room temperature. Then the solution was treated with ammonia solution to pH 9-10 and an orange precipitate was formed. The precipitate was filtred and washed with water. 2-methylbenzimidazolium chloride was gained in 27.32% yield. The compound 2-methylbenzimidazole nitrate was obtained in 35% yield when the 2-methylbenzimidazolium chloride (0.46 g, 2.73 mmol) was reacted with Cr(NO_3_)_3_.9H_2_O (1.01 g, 2.54 mmol) in ethanol under reflux. The crystals suitable for X-ray diffraction analysis were obtained by recrystallization from ethanol.

### Refinement

All H atoms were located in difference maps. H atoms bonded to C atoms were then treated as riding atoms in geometrically idealized positions, with C—H distances of 0.93 (aromatic), 0.96 (CH3—H) and 0.86 (N—H) Å, and with U_iso_(H) =kU_eq_(C), where k is 1.5 for the methyl group and 1.2 for all the other H atoms.

### Crystal data

**Table:**

C_8_H_9_N_2_^+^·NO_3_^−^	*F*(000) = 408
*M**_r_* = 195.18	*D*_x_ = 1.362 Mg m^−^^3^
Monoclinic, *P*2_1_/*c*	Mo *K*α radiation, λ = 0.71073 Å
Hall symbol: -P 2ybc	Cell parameters from 2174 reflections
*a* = 7.711 (4) Å	θ = 2.5–25.5°
*b* = 15.127 (7) Å	µ = 0.11 mm^−^^1^
*c* = 8.270 (4) Å	*T* = 298 K
β = 99.398 (7)°	Block, colorless
*V* = 951.7 (8) Å^3^	0.18 × 0.16 × 0.12 mm
*Z* = 4	

### Data collection

**Table:**

Bruker SMART APEX diffractometer	1685 independent reflections
Radiation source: fine-focus sealed tube	1319 reflections with *I* > 2σ(*I*)
graphite	*R*_int_ = 0.027
φ and ω scans	θ_max_ = 25.1°, θ_min_ = 2.7°
Absorption correction: multi-scan (*SADABS*; Bruker, 2005)	*h* = −9→9
*T*_min_ = 0.981, *T*_max_ = 0.987	*k* = −11→18
4774 measured reflections	*l* = −9→9

### Refinement

**Table:**

Refinement on *F*^2^	Primary atom site location: structure-invariant direct methods
Least-squares matrix: full	Secondary atom site location: difference Fourier map
*R*[*F*^2^ > 2σ(*F*^2^)] = 0.045	Hydrogen site location: inferred from neighbouring sites
*wR*(*F*^2^) = 0.135	H-atom parameters constrained
*S* = 1.07	*w* = 1/[σ^2^(*F*_o_^2^) + (0.0681*P*)^2^ + 0.208*P*] where *P* = (*F*_o_^2^ + 2*F*_c_^2^)/3
1685 reflections	(Δ/σ)_max_ < 0.001
128 parameters	Δρ_max_ = 0.22 e Å^−^^3^
0 restraints	Δρ_min_ = −0.21 e Å^−^^3^

### Special details

**Table:**

Geometry. All esds (except the esd in the dihedral angle between two l.s. planes) are estimated using the full covariance matrix. The cell esds are taken into account individually in the estimation of esds in distances, angles and torsion angles; correlations between esds in cell parameters are only used when they are defined by crystal symmetry. An approximate (isotropic) treatment of cell esds is used for estimating esds involving l.s. planes.
Refinement. Refinement of *F*^2^ against ALL reflections. The weighted *R*-factor wR and goodness of fit *S* are based on *F*^2^, conventional *R*-factors *R* are based on *F*, with *F* set to zero for negative *F*^2^. The threshold expression of *F*^2^ > σ(*F*^2^) is used only for calculating *R*-factors(gt) etc. and is not relevant to the choice of reflections for refinement. *R*-factors based on *F*^2^ are statistically about twice as large as those based on *F*, and *R*- factors based on ALL data will be even larger.

### Fractional atomic coordinates and isotropic or equivalent isotropic displacement parameters (Å^2^)

**Table:**

	*x*	*y*	*z*	*U*_iso_*/*U*_eq_	
O1	0.3641 (2)	0.06287 (10)	0.78462 (18)	0.0735 (5)	
O2	0.33057 (19)	0.15524 (9)	0.97555 (17)	0.0643 (4)	
O3	0.2248 (3)	0.02349 (11)	0.9773 (2)	0.0915 (6)	
N1	0.3063 (2)	0.07987 (10)	0.9136 (2)	0.0538 (4)	
N2	0.6679 (2)	0.12445 (11)	0.25067 (19)	0.0600 (5)	
H2	0.6828	0.0687	0.2374	0.072*	
N3	0.5577 (2)	0.24731 (11)	0.31726 (19)	0.0576 (5)	
H3	0.4889	0.2842	0.3542	0.069*	
C1	0.3941 (3)	0.11166 (15)	0.3743 (3)	0.0727 (6)	
H1A	0.3921	0.0516	0.3368	0.109*	
H1B	0.2839	0.1395	0.3331	0.109*	
H1C	0.4132	0.1126	0.4920	0.109*	
C2	0.5376 (3)	0.15990 (13)	0.3145 (2)	0.0569 (5)	
C3	0.7769 (3)	0.19049 (13)	0.2083 (2)	0.0561 (5)	
C4	0.7057 (3)	0.26993 (13)	0.2519 (2)	0.0542 (5)	
C5	0.7834 (3)	0.35057 (14)	0.2303 (3)	0.0677 (6)	
H5	0.7356	0.4035	0.2595	0.081*	
C6	0.9355 (3)	0.34816 (19)	0.1630 (3)	0.0805 (7)	
H6	0.9920	0.4009	0.1463	0.097*	
C7	1.0070 (3)	0.2684 (2)	0.1191 (3)	0.0802 (7)	
H7	1.1095	0.2695	0.0734	0.096*	
C8	0.9302 (3)	0.18829 (18)	0.1416 (3)	0.0715 (7)	
H8	0.9787	0.1353	0.1134	0.086*	

### Atomic displacement parameters (Å^2^)

**Table:**

	*U*^11^	*U*^22^	*U*^33^	*U*^12^	*U*^13^	*U*^23^
O1	0.1006 (12)	0.0560 (9)	0.0718 (10)	0.0061 (8)	0.0370 (9)	−0.0048 (7)
O2	0.0865 (10)	0.0434 (8)	0.0651 (9)	−0.0085 (7)	0.0188 (7)	−0.0067 (6)
O3	0.1307 (16)	0.0554 (10)	0.0990 (13)	−0.0287 (10)	0.0499 (12)	0.0017 (8)
N1	0.0628 (10)	0.0406 (9)	0.0590 (10)	0.0020 (7)	0.0127 (8)	0.0042 (7)
N2	0.0735 (11)	0.0464 (9)	0.0564 (10)	0.0153 (8)	−0.0006 (8)	−0.0086 (7)
N3	0.0710 (11)	0.0476 (10)	0.0521 (9)	0.0150 (8)	0.0038 (8)	−0.0094 (7)
C1	0.0902 (16)	0.0612 (14)	0.0673 (13)	0.0017 (12)	0.0145 (12)	−0.0006 (11)
C2	0.0728 (13)	0.0487 (12)	0.0462 (10)	0.0126 (10)	0.0004 (9)	−0.0052 (8)
C3	0.0621 (12)	0.0575 (12)	0.0440 (10)	0.0131 (10)	−0.0051 (8)	−0.0086 (9)
C4	0.0619 (11)	0.0536 (11)	0.0433 (10)	0.0096 (9)	−0.0024 (8)	−0.0058 (8)
C5	0.0810 (15)	0.0539 (13)	0.0631 (13)	0.0037 (11)	−0.0033 (11)	−0.0019 (10)
C6	0.0806 (16)	0.0842 (18)	0.0716 (15)	−0.0133 (14)	−0.0030 (12)	0.0061 (13)
C7	0.0679 (14)	0.105 (2)	0.0662 (14)	0.0061 (14)	0.0070 (11)	−0.0032 (14)
C8	0.0685 (14)	0.0831 (17)	0.0593 (13)	0.0169 (13)	−0.0007 (11)	−0.0124 (11)

### Geometric parameters (Å, °)

**Table:**

O1—N1	1.248 (2)	C1—H1C	0.9600
O2—N1	1.252 (2)	C3—C8	1.384 (3)
O3—N1	1.228 (2)	C3—C4	1.393 (3)
N2—C2	1.322 (3)	C4—C5	1.383 (3)
N2—C3	1.387 (3)	C5—C6	1.378 (4)
N2—H2	0.8600	C5—H5	0.9300
N3—C2	1.331 (3)	C6—C7	1.399 (4)
N3—C4	1.383 (3)	C6—H6	0.9300
N3—H3	0.8600	C7—C8	1.374 (4)
C1—C2	1.476 (3)	C7—H7	0.9300
C1—H1A	0.9600	C8—H8	0.9300
C1—H1B	0.9600		
			
O3—N1—O1	120.19 (17)	C8—C3—N2	132.5 (2)
O3—N1—O2	120.66 (17)	C8—C3—C4	121.5 (2)
O1—N1—O2	119.14 (16)	N2—C3—C4	105.96 (18)
C2—N2—C3	109.90 (17)	C5—C4—N3	132.13 (19)
C2—N2—H2	125.1	C5—C4—C3	122.0 (2)
C3—N2—H2	125.1	N3—C4—C3	105.85 (18)
C2—N3—C4	109.90 (16)	C6—C5—C4	116.3 (2)
C2—N3—H3	125.1	C6—C5—H5	121.8
C4—N3—H3	125.1	C4—C5—H5	121.8
C2—C1—H1A	109.5	C5—C6—C7	121.7 (2)
C2—C1—H1B	109.5	C5—C6—H6	119.2
H1A—C1—H1B	109.5	C7—C6—H6	119.2
C2—C1—H1C	109.5	C8—C7—C6	121.9 (2)
H1A—C1—H1C	109.5	C8—C7—H7	119.1
H1B—C1—H1C	109.5	C6—C7—H7	119.1
N2—C2—N3	108.40 (19)	C7—C8—C3	116.6 (2)
N2—C2—C1	126.37 (19)	C7—C8—H8	121.7
N3—C2—C1	125.23 (19)	C3—C8—H8	121.7
			
C3—N2—C2—N3	−0.6 (2)	C8—C3—C4—N3	−178.53 (17)
C3—N2—C2—C1	179.86 (19)	N2—C3—C4—N3	−0.24 (19)
C4—N3—C2—N2	0.4 (2)	N3—C4—C5—C6	178.52 (19)
C4—N3—C2—C1	179.99 (19)	C3—C4—C5—C6	0.0 (3)
C2—N2—C3—C8	178.5 (2)	C4—C5—C6—C7	0.0 (3)
C2—N2—C3—C4	0.5 (2)	C5—C6—C7—C8	−0.4 (4)
C2—N3—C4—C5	−178.8 (2)	C6—C7—C8—C3	0.7 (3)
C2—N3—C4—C3	−0.1 (2)	N2—C3—C8—C7	−178.4 (2)
C8—C3—C4—C5	0.3 (3)	C4—C3—C8—C7	−0.7 (3)
N2—C3—C4—C5	178.59 (17)		

### Hydrogen-bond geometry (Å, °)

**Table:**

*D*—H···*A*	*D*—H	H···*A*	*D*···*A*	*D*—H···*A*
N2—H2···O1^i^	0.86	2.03	2.855 (3)	162
N3—H3···O2^ii^	0.86	1.93	2.775 (2)	166

Symmetry codes: (i) −*x*+1, −*y*, −*z*+1; (ii) *x*, −*y*+1/2, *z*−1/2.
